# Analysis of protein profile expressed on the membrane surface of plasma cells, stromal cells and mononuclear cells from bone marrow microenvironment of multiple myeloma patients by mass spectrometry

**DOI:** 10.1186/1753-6561-7-S2-P23

**Published:** 2013-04-04

**Authors:** Fabricio de Carvalho, Adriana FP Leme, Alisson L Matsuo, Sandra S Matsuda, Bianca A Pauletti, Walter MT Braga, Edgar J Paredes-Gamero, Maria de Lourdes LF Chauffaille, Gisele WB Colleoni

**Affiliations:** 1 Department of Clinical and Experimental Oncology, Hematology and Hemotherapy, Universidade Federal de São Paulo, UNIFESP/EPM, São Paulo, Brazil; 2 Mass Spectrometry Laboratory, Bioscience Laboratory, CNPEM/ABTLuS, Campinas, Brazil; 3 Department of Microbiology, Immunology and Parasitology, Cell Biology, UNIFESP/EPM, São Paulo, Brazil; 4 Department of Biochemistry, Molecular Biology, UNIFESP/EPM, São Paulo, Brazil

## Background

Multiple Myeloma (MM) is a malignant proliferation of B-cells with plasma cell differentiation. Interactions of plasma cells with stromal cells and other cells in bone marrow (BM) microenvironment are responsible for MM progression. Screening of proteins expressed on the membrane surface of plasma cells, stromal cells and mononuclear cells from MM patients by mass spectrometry (MS) may identify new prognostic and therapeutic targets in this incurable disease.

## Materials and methods

BM from eight MM patients, five normal BM from donors and seven tonsils of children submitted to tonsillectomy (controls) were collected. Plasma cells (CD138+) from MM patients and tonsils, stromal cells (CD105+) and mononuclear cells from MM patients and normal BM were isolated by magnetic cell sorting (MACS). Stromal cells were *in vitro* expanded and characterized by immunocytochemistry and flow cytometry (CD105, Vimentin, CD45 and cytokeratin [CK]). Proteins were obtained by Pierce Cell Surface Protein Isolation kit and quantified by Pierce 660nm Protein Assay.

## Results and conclusion

*In vitro* expansion of stromal cells was possible for four MM and four normal BM samples. Stromal cells were CD105+, Vimentin+, CD45- and CK-, without contamination with other cell types. Plasma cell pool from MM patients or tonsils (control), stromal cell pool and mononuclear cell pool from MM patients or normal BM (control) were subjected to protein extraction (yield ranged from 50μg to 100μg). Analysis of proteins will be performed by LTQ (Linear Ion Trap Quadropole) Orbitrap Velos Mass Spectrometry and the spectra MS/MS will be obtained using Mascot.

## Financial support

São Paulo Research Foundation (FAPESP), Brazil.

